# Evolution, Prehistory and Vitamin D

**DOI:** 10.3390/ijerph17020646

**Published:** 2020-01-19

**Authors:** Paul Jarrett, Robert Scragg

**Affiliations:** 1 Department of Dermatology, Middlemore Hospital, Auckland 2025, New Zealand; 2 Department of Medicine, The University of Auckland, Auckland 1023, New Zealand; 3 Department of Population Health, The University of Auckland, Auckland 1072, New Zealand; r.scragg@auckland.ac.nz

**Keywords:** evolution, folate, migration, human, Africa, clothing, louse, technology, vitamin D

## Abstract

Aspects of human evolutionary biology and prehistory are discussed in relation to vitamin D. The evolution of hairlessness, combined with the need for efficient eccrine sweat production for cooling, provided evolutionary pressure to protect the skin from ultraviolet damage by developing cutaneous pigmentation. There was a subsequent loss of pigmentation as humans journeyed to northern latitudes. Their increasing mastery of technology outstripped evolution’s finite pace as further dispersal occurred around the globe. A timeline for the development of clothing to provide warmth, and the consequent shielding from ultraviolet light, which diminished vitamin D synthesis, can be inferred by an examination of mutations in the human louse.

## 1. Introduction

In the modern world, it is easy to take for granted the speed and ease of travel and accept the accelerating pace of change in contemporary society whilst forgetting the role of evolutionary biology on our prehistorical ancestors and its persisting effects in relation to vitamin D. Evolutionary changes in biological systems have finite speed, but the rapid mastery of technology by humans far outstrips the evolutionary process. For example, consider Moore’s law of the exponential growth in the complexity of computer integrated circuits proposed just 50 years ago and the potential for quantum computing. In contrast to such technological advances, the evolutionary story starts millions of years ago. This review considers some of the evolutionary changes in the structure and function of skin during prehistory in relation to vitamin D.

## 2. Evolution of Skin and Vitamin D

The generally accepted view is that modern humans (*Homo sapiens*) originated from Africa [1,2]. The earliest members of our lineage were members of the genus *Ardipithecus* and lived mostly in woodland environments [3]. Later protohumans, also referred to as hominids, in the genus *Australopithecus*, lived in a wider range of environments but have also only been recovered from Africa [4]. Data from fossil records to study climate change indicate that approximately 2.6 million years ago, as the Pleistocene Epoch began, the habitat of protohumans changed as the earth entered the period of climatic oscillations known as the ice ages. Perhaps in response to the increased variance in the environment, approximately 2.6 million years ago the archaeological evidence indicates that our ancestors started to use stone tools to butcher animal bones [5]. Meat is a richer source of nutrients than vegetable matter but it is scarce and mobile. In addition to the stone tools, these early members of the genus *Homo* also evolved modern human-like skeletal proportions that reflect a more efficient long-distance walking ability [6]. With the greater energy expenditure of daytime long-distance walking came the need for greater thermal regulation, particularly cooling. Bipedalism carries significant competitive advantages of speed, height, and the use of tools. *Homo erectus*, 1.6 million years ago, was the first hominid to have elongated limbs capable of sustained walking and running. Therefore, the switch from less hair to an efficient cooling system was underway 1.6 million years ago [7].

There is an evolutionary advantage to hairlessness. The modern-day chimpanzee is our closest living relative. Chimpanzees have pink skin covered with black fur. The hominids are believed to have shared this phenotype. Hair provides effective sun protection and thermoregulation for a sedentary lifestyle. The most efficient evaporative cooling of sweat occurs at the skin’s surface and then water vapour is transferred through the fur. Dry fur also protects the body from external environmental heat gain. If fur becomes wet, evaporation occurs at the surface of the fur and not at the skin, and therefore heat from the cutaneous vessels has a barrier to its site of loss. Consequently, there is an advantage to hairlessness when there is a need for significant heat loss, which will occur with exercise [8]. 

In modern humans, there are three types of sweat glands in the skin: eccrine, apocrine, and apoeccrine. They secrete fluid directly into the duct. These glands vary in type, density, and anatomical location. The eccrine glands are the most important for temperature control. Approximately 1.6 to 4 million are distributed over most of the body’s surface. Eccrine sweat is a sterile dilute electrolyte solution. The apocrine glands are limited in their distribution to axillae, anogenital, and periumbilical skin, nipples, and vermillion border and they connect by a stretched duct into the follicular canal. Apocrine sweat is a sterile viscous oily fluid. The apoeccrine glands are confined to the adult axilla. 

The chimpanzee, gorilla, and baboon have fur coats. These animals have thermal apocrine glands and eccrine glands. Therefore, it is inferred that the ancestral great apes were able to “supply” eccrine glands to the protohumans. These coats provide physical protection and are efficient cooling systems, using apocrine sweat when dry, but are not effective cooling systems when wet from sweat production. Thus, hair is disadvantageous for heat loss after prolonged physical exercise. There are no thermal apocrine glands that are not associated with hair follicles. Natural selection, therefore, drove the loss of hair and apocrine glands to favour the development of eccrine sweating for effective temperature control in concert with bipedalism [8].

The central equatorial African savannah is a high ultraviolet (UV) environment, and a lack of hair results in UV-induced damage through a lack of sun protection. The effect of climate change producing a drier environment also placed stress on the epidermal barrier. Therefore, evolutionary pressures worked to provide the needed protection, and there are several theories concerning this, including the need to protect against UV induced skin cancer and UV induced barrier dysfunction as well as the requirement for folic acid protection. Normal functioning of the skin barrier with an intact epidermis is essential for health, and atopic dermatitis is an example of a disease with disrupted function. Pigmented skin has enhanced barrier function, with greater cohesion of the stratum corneum and reduced susceptibility to infection, as well as providing protection from UV induced dysplasia and skin cancers [9]. 

Folic acid is an essential vitamin needed for numerous biological functions, including DNA synthesis and repair, red blood cell production and spermatogenesis. In humans, disorders associated with lack of folic acid include megaloblastic anaemia, peripheral neuropathy, and, in pregnancy, foetal neural tube defects. Folic acid deficiency is also associated with multiple defects in non-human mammals. In the human body, folate (the naturally occurring form) is sensitive to UV-induced degradation in the skin. UV-induced folate degradation has been demonstrated in light-skinned patients exposed to natural sunlight. Therefore, there was a strong evolutionary pressure to protect the skin and this was achieved by the production of melanin [10]. 

Melanin is produced in the melanocyte. The melanocyte is a cell derived from the neural crest that, in the skin, resides in the basal layer of the epidermis. The production of melanin is complex and occurs in the intracytoplasmic organelle called the melanosome. This moves along the dendritic process of the melanocyte and is then transferred to the keratinocyte. It is the activity, and not the number of melanocytes that determines skin colour. Darkly pigmented skin has melanosomes with a heavy deposition of melanin compared with fair skin, which has minimal melanin deposition. There are two major forms of melanin produced by melanocytes: brown-black eumelanin and yellow-red phaeomelanin. Melanin attenuates UV radiation by absorption, dissipating it as heat. The melanocortin 1 receptor (MC1R) is a membrane-bound receptor on melanocytes and is one of the most important regulators of melanin production [11].

The *MC1R* gene is mapped to chromosome 16q24. There is almost no variation in this coding region in African populations, supporting the strong selective pressure to maintain a dark skin colour in the African environment [12,13]. Therefore, at some point in the transition from the hairy to the hairless state, evolutionary pressure would have acted to support the selection of the MC1R alleles producing skin pigmentation. Genetic modelling suggests that this gene variant found in Africans may have emerged 1.2 million years ago, which roughly corresponds to an innovation in stone tool technology that likely reflects an increase in the sophistication of hunting ability [14,15].

The vitamin D binding protein (VDBP) has also been subject to evolutionary pressure that can be considered a continuous process of structural modification from primates [16]. The gene was the target of locally exerted selective pressure driving different haplotypes in distinct human populations [17]. There are different polymorphisms of the VDBP, with group-specific component (GC) 1F being most abundant in persons of African ancestry and GC1S being most abundant in European populations [18]. The affinity of the two VDBPs is different for vitamin D, with GC1F being greater than GC1S, and it is possible that during evolution the most abundant form of the VDBP in dark skin was able to transport vitamin D_3_ more efficiently from the skin to the liver for its metabolism to 25-hydroxyvitamin D [19]. There are lower levels of 25-hydroxyvitamin D in the African-American population, but there are higher bone densities in the African-American population compared with that of the white population. Lower levels of VDBP in African-Americans will result in bioavailable levels of 25-hydroxyvitamin D equivalent to those in caucasians [20]. However, the methodology and therefore the conclusions of this study have been challenged for a number of reasons, including not considering the role of the renal proximal tubule, the methodology of calculating the bioavailable 25-hydroxyvitamin D, and the monoclonal antibodies used [21,22].

## 3. Prehistory and Vitamin D

The exact method, mode, and timing of human dispersion out of central Africa are uncertain, but the older archaeological evidence of this migration is supported by a modern genomic analysis of ancient bones [2,23]. Human dispersal from Africa is unlikely to have occurred in one wave and is more likely to have occurred in multiple waves with the following two major episodes: the first through the Arab peninsula into southern Asia and Oceania and a later wave through a northern route [24]. These uncertainties aside, humans walked north probably through modern-day Egypt and the eastern Mediterranean into Europe and east into Asia, perhaps following the coastline [25]. There have been numerous changes in coast lines [26] and the ancient coastline was different from today. Sea levels were lower because of large quantities of water locked in polar ice caps. The migration of protohumans in eastern Asia was occurring during the Pleistocene era (2.6 million to 11,700 years ago) [27]. Movement further east into the Pacific may have occurred from Taiwan (the “out of Taiwan model”) or possibly from Wallacea, which is a geographical group of islands between the Asian and Australian continental shelves. However, recent genome-wide data from 56 Austronesian groups suggest that their ancestry is closely related to aboriginal Taiwanese favouring the “out of Taiwan model” [28]. The last major migration of humans was into the remote eastern Pacific during the Holocene era (10,000 years ago to current) [29]. The Pacific rat (*Rattus exulans*) travelled with ancient humans and can be used as a proxy to estimate the time of arrival at a given location. Radiocarbon dating of distinctive rat-gnawed seeds and rat bones from different locations around New Zealand show that the rat was established in New Zealand by approximately 1280 in the common era, and there is no evidence to suggest the presence of rats during the preceding millennium [30]. This time frame is supported by mitochondrial DNA studies of Māori, whose founder population of women arrived in the sea-going waka (canoes) numbering 170–230 [31].

As humans migrated away from the central equatorial African climate to different latitudes, the exposure to ultraviolet B (UVB) diminished and therefore the ability to produce sufficient vitamin D also diminished. Highly melanised skin requires longer exposure to UVB when the intensity is reduced in order to produce sufficient vitamin D. Rickets is a serious complication of vitamin D deficiency which would have resulted in deformed pelvises that were inadequate for successful childbirth in prehistory if the evidence of recent history is reviewed. In 1956, approximately 15% of African-American women had significantly deformed pelvises due to childhood vitamin D deficiency, which reflected the cohort of women born prior to the widespread adoption of dietary vitamin D supplements in the 1930s [32]. The obstetric complications included abnormal presentation, umbilical cord prolapse, and impossible vaginal delivery in 10% of cases, with foetal mortality at 10–15% in the absence of caesarean section [32]. Therefore, evolutionary selective pressure favoured the loss of melanin over tens of thousands of years. Depigmentation was evolved through different complex genetic mechanisms in northern Europeans, modern East Asians and Neanderthal humans [33,34,35,36,37].

There is a strong correlation of skin reflectance with latitude and UV radiation. Interestingly, in all the studied populations, females are found to have lighter skin than males. The lighter skin of females may be due to the evolutionary pressure to produce greater quantities of vitamin D during pregnancy and lactation [10]. New Zealand lies approximately between latitude 35° and 46° south. Wellington sits at 41°19′ south. The potential for the synthesis of previtamin D_3_ in the skin has been estimated from average annual UV minimal erythemal doses. The minimal erythemal dose is the quantity of UV radiation required to produce a barely perceptible reddening of the skin. New Zealand and southern Australia, including Tasmania, fall into a zone where there is insufficient UV radiation to catalyse the formation of previtamin D_3_ in moderately and highly melanised skin. In lightly pigmented skin, these areas fall into the category for which there is insufficient UV radiation during at least one month of the year to produce vitamin D_3_ [10]. Therefore, approximately 700 years ago, the earliest Polynesian colonisers of New Zealand, arriving by boat, entered a UV environment, setting the stage for relative vitamin D deficiency. This is an example of how the technology of the day, the sea-going waka, outstripped evolution. In New Zealand, Māori women have significantly lower levels of vitamin D than non-Māori women, and Pacific men and women have significantly lower levels of vitamin D than non-Pacific men and women [38]. Modern migrants travelling to New Zealand arrive by plane from all over the world and those with low levels of vitamin D, including those of Indian and African descent but those of Asian ethnicity have the lowest mean level (37.0 nmol/L) [39,40].

Although diet is important, 90% of vitamin D synthesis is derived from cutaneous sun exposure [41,42]. Therefore, the development of clothing, which blocks UV exposure, is important. Protection from cold is a leading theory for the development of clothing [43] particularly relevant in the higher and lower northern and southern latitudes of the globe where the temperatures are cooler, the need for warmth greater and the incident UV is less than that in central equatorial latitudes. The development of clothing by ancient humans is another example of adoption of the use of technologies that include scraping, cutting pelts, and the ability to pierce and sew, which has the unintended consequence of compounding potential vitamin D deficiency. In the archaeological record, needles and hide scrapers can be found but it is likely that the use of clothing predated the development of this technology as ancient clothing would degrade quickly. However, the study of human lice *Pediculosus humanus capitis* (head lice) and *Pediculosis humanus humanus* (body lice) has shed light on the first use of clothing. These two lice are morphologically similar but inhabit different body sites. Head lice live on the scalp and feed more frequently than the body lice that live in clothing and move to the body to feed once or twice a day [44]. Sucking lice parasitising mammals are generally host specific [45], and body lice are derived from head lice [46]. With human evolution and the loss of body hair, the louse needed to evolve and diverged in to the two types [47]. All modern body lice associated with modern humans are confined to a single mitochondrial descendant (clade) [47]. Examining when that divergence took place provides a proxy estimate as to when humans may have started to wear clothing and it is estimated that this divergence may have occurred between 83,000 and 170,000 years ago during the middle to late Pleistocene era [47]. If this is correct, then vitamin D deficiency may be a relatively modern phenomenon in the evolutionary timeline. It is notable that the Neolithic (12,000–3500 years before the common era) skeleton of a 25–30 year-old female found on the Scottish island of Tiree (latitude 56.5° north) had deformation of the sternum and ribs consistent with rickets [48].

## 4. Conclusions

Human imagination, combined with the ability to develop technology, such as tools, clothing, and sea-going waka, far outpace biological processes, however, our evolutionary roots still have a direct consequence onvitamin D in modern society.
